# A RNase P Ribozyme Inhibits Gene Expression and Replication of Hepatitis B Virus in Cultured Cells

**DOI:** 10.3390/microorganisms11030654

**Published:** 2023-03-03

**Authors:** Bin Yan, Yujun Liu, Yuan-Chuan Chen, Fenyong Liu

**Affiliations:** 1 School of Public Health, University of California, Berkeley, CA 94720, USA; 2 Program in Comparative Biochemistry, University of California, Berkeley, CA 94720, USA

**Keywords:** RNase P, ribozyme, hepatitis B virus, antisense, antiviral, gene targeting, gene therapy

## Abstract

Hepatitis B virus (HBV), an international public health concern, is a leading viral cause of liver disease, such as hepatocellular carcinoma. Sequence-specific ribozymes derived from ribonuclease P (RNase P) catalytic RNA are being explored for gene targeting applications. In this study, we engineered an active RNase P ribozyme, M1-S-A, targeting the overlapping region of HBV S mRNA, pre-S/L mRNA, and pregenomic RNA (pgRNA), all deemed essential for viral infection. Ribozyme M1-S-A cleaved the S mRNA sequence efficiently in vitro. We studied the effect of RNase P ribozyme on HBV gene expression and replication using the human hepatocyte HepG2.2.15 culture model that harbors an HBV genome and supports HBV replication. In these cultured cells, the expression of M1-S-A resulted in a reduction of more than 80% in both HBV RNA and protein levels and an inhibition of about 300-fold in the capsid-associated HBV DNA levels when compared to the cells that did not express any ribozymes. In control experiments, cells expressing an inactive control ribozyme displayed little impact on HBV RNA and protein levels, and on capsid-associated viral DNA levels. Our study signifies that RNase P ribozyme can suppress HBV gene expression and replication, implying the promise of RNase P ribozymes for anti-HBV therapy.

## 1. Introduction

Nucleic acid-based gene interfering molecules, such as antisense oligonucleotides, ribozymes, and small interfering RNA (siRNA), represent exciting gene-targeting agents [1]. Ribozymes derived from ribonuclease P (RNase P) catalytic RNAs are being explored as potential gene-targeting agents. RNase P is a ribonucleoprotein complex enzyme catalyzing the removal of the precursor tRNA leader sequence during tRNA maturation [2,3,4]. RNase P from *E. coli* comprises of a protein subunit, C5 protein, and a catalytic RNA subunit, M1 RNA [5,6]. Altman and colleagues showed RNase P and M1 RNA can be employed to cleave any mRNA using a custom-designed external guide sequence (EGS) to hybridize with a specific mRNA target, forming a structure which resembles a tRNA substrate [7,8]. Moreover, a ribozyme, M1GS, can be engineered to be an mRNA-targeting endoribonuclease by attaching to M1 RNA a guide sequence complementary to an mRNA. M1GS ribozymes have been shown to cleave numerous mRNA sequences in vitro and suppress their expression in cultured cells. These ribozymes were also able to suppress the infection and replication of murine cytomegalovirus, human cytomegalovirus, and HIV in cultured cells [9,10,11].

Hepatitis B virus (HBV), with more than 400 million chronic infections globally, is a prominent viral cause of hepatic disease [12,13,14,15]. Hepatocytes are the site for HBV infection, which can result in severe diseases such as cirrhosis and hepatocellular carcinoma [13,15,16]. Successful treatment of HBV-associated diseases requires suppression of HBV infection in hepatocytes.

While current measures such as a preventative vaccine are available, the reality is that current FDA-approved therapy side-effect profiles, together with the advent of drug-resistant HBV strains [13,15], necessitate the ongoing expansion of novel compounds and methods in the treatment of HBV-positive individuals. Nucleic acid-based gene-interfering molecules, such as hammerhead ribozymes and small interfering RNAs (siRNA), have been explored as anti-HBV agents for inhibiting HBV gene expression and replication both in vitro and in vivo [17,18,19,20]. Whether ribozymes derived from a RNase P catalytic RNA can be constructed to achieve anti-HBV activity, however, has not been reported.

In the present study, we engineered M1GS ribozymes with specificity against the overlapping region (S RNA) of the S mRNA, pre-S/L mRNA, and pregenomic RNA (pgRNA) of HBV, which are essential for HBV replication and infection [15]. We demonstrated that the active RNase P ribozyme, M1-S-A, can slice the S mRNA sequence in vitro. When expressed in cells, M1-S-A substantially inhibited the levels of HBV RNA transcripts, proteins, and genomic DNA, compared to the HBV-infected cells not expressing any ribozymes. In control experiments, little suppression was noted in cells expressing a control inactive ribozyme that was able to bind the S RNA target sequence as well as M1-S-A but was not catalytically active due to mutations at the ribozyme active domain. Our results provide the first direct evidence that M1GS RNAs suppress HBV gene expression and replication. These results also reveal the feasibility of producing RNase P ribozymes for anti-HBV therapy.

## 2. Materials and Methods

### 2.1. Cells and Dimethyl Sulphate-Mediated Mapping of Viral RNAs in Cells

HepG2 (American Type Culture Collection, Manassas, VA, USA) and HBV-infected HepG2.2.15 cells were maintained in Dulbecco’s modified Eagle medium (DMEM) supplemented with 10% heat-inactivated fetal calf serum as defined previously [21,22]. HepG2.2.15 cells were mixed with fresh media containing 1% of dimethyl sulphate (DMS) for 5–10 min, and then washed 3 times with phosphate-buffered saline (PBS) that contained 1 mM β–mercaptoethanol, and lysed by adding cell lysis buffer (150 mM NaCl, 10 mM Tris-HCl pH 7.4, 1.5 mM MgCl_2_, 0.2% NP40). We purified total RNAs by phenol-chloroform extraction and ethanol precipitation and then performed primer extension assays in the presence of reverse transcriptase and [^32^P]-labeled oligonucleotide primers specific for the S mRNA following protocols described previously [23,24]. Primer extension products were separated in urea-containing denaturing polyacrylamide gels and analyzed with a STORM840 Phosphorimager to identify the DMS-modified regions of the S mRNA [23,24].

### 2.2. Studies In Vitro of the M1GS RNAs and S mRNA Substrate

The DNA sequence coding for HBV substrate s37 was constructed by PCR using pGEM3zf(+) as a template and oligonucleotides AF25 (5′-GGAATTCTAATACGACTCACTATAG-3′) and s37-3 (5′-CGGGATCCGTTCCTGGAAGTAGAGGACAAACGGGCAATCTATAGTGAGTCGTATTA-3′) as 5′ and 3′ primers, respectively.

Plasmid pFL117 and pC102 contain the DNA sequence coding for active ribozyme M1 RNA and inactive mutant ribozyme C102 driven by the T7 RNA polymerase promoter [25,26]. Plasmid pM1-P contains the DNA sequence coding for active ribozyme M1-P targeting the mRNA which encodes the essential protease of human cytomegalovirus (HCMV) [10]. Ribozyme C102 was derived from M1 RNA and contains several point mutations (e.g., A_347_C_348_ -> C_347_U_348_, C_353_C_354_C_355_G_356_ -> G_353_G_354_A_355_U_356_) at the catalytic domain (P4 helix) [26]. The DNA sequences coding for ribozymes M1-S-A and M1-S-I were constructed by PCR using the DNA sequences of the ribozymes as the templates and oligonucleotides AF25 and Rb-S-3 (5′-CCCGCTCGAGAAAAAATGGTGTCCTCTACTTCCAGGAATGTGGAATTGTG-3′) as 5′ and 3′ primers, respectively. Synthesis of substrate s37 and ribozymes M1-S-A, M1-S-I, and M1-P was performed in vitro with the T7 RNA polymerase-mediated transcription system.

For the binding between M1GSs and substrate s37, assays were performed in vitro in buffer E (50 mM Tris, pH 7.5; 100 mM NH_4_Cl, 100 mM CaCl_2_, 3% glycerol, 0.1% xylene cyanol, 0.1% bromophenol blue) [25,27,28]. The binding complexes were separated on 6% polyacrylamide non-denaturing gels [29]. The values of K_d_ were the averages from three independent experiments [25,27,28]. We also performed the assays for the cleavage of s37 by ribozymes in vitro in buffer A (50 mM Tris, pH 7.5; 100 mM NH_4_Cl, 100 mM MgCl_2_), following procedures noted previously [29,30,31]. The cleavage products were separated on 8% polyacrylamide denaturing gels. The values of the overall cleavage rate (k_cat_/K_m_)^s^ were determined from triplicate experiments [25,27,28].

### 2.3. Cell Line Construction for Ribozyme Expression

Constructs pU6-M1-S-A, pU6-M1-S-I, and pU6-M1-P were generated by inserting the DNA sequences coding for ribozyme M1-S-A, M1-S-I, and M1-P into the expression vector pU6. Following the experimental procedures described previously [30,32], HepG2.2.15 cells were transfected with pU6-M1-S-A, pU6-M1-S-I, pU6-M1-P, and empty vector pU6, and subsequently selected and cloned in the presence of 600 µg/mL neomycin (Invitrogen, Carlsbad, CA, USA). Total RNAs were isolated from cells using Trizol (Invitrogen, San Diego, CA, USA) and digested with DNase I [8,21,30,33]. Using radiolabeled probes complementary to M1 RNA and H1 RNA, we performed Northern blot analyses to assess the M1GS ribozyme expression with H1 RNA expression as the loading control [34,35].

### 2.4. Cytotoxicity Assays

M1GS expression-associated cytotoxicity was assessed by an MTT assay (Sigma, St Louis, MO, USA). We grew cells in 96-well plates carrying empty vector construct pU6 and ribozyme-containing constructs (i.e., pU6-M1-S-A, pU6-M1-S-I, pU6-M1-P). At different time points, we added 3-(4,5-Dimethylthiazol-2-yl)-2,5-diphenyl tetrazolium bromide (MTT; Sigma) (5 mg/mL in PBS) to each well and determined cell viability following the manufacturer’s recommendations [36]. We then used a microplate reader to assay the absorbance at 570 nm. Moreover, we employed a Nikon TE300 microscope to monitor cell morphology at different time points.

### 2.5. Determination of HBV RNA and Protein Levels

RNA and protein samples were isolated with previously defined methods [8,21,30,33]. Proteins in cultured cells and media were assayed by enzyme-linked immunosorbent assay (ELISA) using the HBsAg or HBeAg diagnostic kits (Abbott Laboratories, Abbott Park, IL, USA; Shanghai KeHua Biotech Co., Shanghai, China), following the manufacturers’ recommendations [21]. Total RNAs were isolated from cells using Trizol (Invitrogen, San Diego, CA, USA), treated with DNase I, and analyzed in Northern blot experiments using probes detecting the H1 and HBV RNAs with a phosphorimager [21,31,33].

### 2.6. Determination of HBV DNA Levels

To isolate capsid-associated viral DNA, cells were lysed in buffer E (50 mM Tris, pH 7.5, 0.5% NP-40, 1 mM EDTA, 100 mM NaCl) and subsequently incubated with DNase I at 37 °C for 6 h [21,31,33]. EDTA (0.5 M) and polyethylene glycol (35% *v*/*v*) were added to the lysate samples, with viral capsids precipitated via centrifugation. The capsid samples were then resuspended in buffer F (10 mM Tris, 100 mM NaCl, 1 mM EDTA, 1% SDS, and 2.5 mg/mL proteinase K) and digested with proteinase K. The viral DNAs were finally purified with phenol and chloroform extraction and ethanol precipitation [21,31,33].

Similar experimental procedures were also used to purify extracellular capsid-associated HBV DNAs in culture media. The culture media were first digested with DNase I in buffer E and then incubated with proteinase K in buffer F to release encapsidated DNA. We finally purified the released HBV DNAs by phenol and chloroform extraction and ethanol precipitation [21,31,33].

Quantitative PCR (qPCR) was utilized to assess capsid-associated DNA levels via an ABI 7500 device (Applied Biosystems Inc., Foster City, CA, USA) or an iCycler Real-Time PCR Detection System (Bio-Rad, Hercules, CA, USA). We employed TaqMan real-time PCR primers including 5′ primer P1 (5′-AGAAACAACACATAGCGCCTCAT-3′), 3′ primer P2 (5′-TGCCCCATGCTGTAGATCTTG-3′), and probe P3 (5′-TGTGGGTCACCATATTCTTGGG-3′) [21]. The PCR reactions were performed as follows: 1 cycle at 50 °C for 2 min, 1 cycle at 95 °C for 10 min, and 40 cycles at 95 °C for 15 s and 60 °C for 60 s. We used plasmid pHBV1.3 as a standard with the dilution range from 10^7^ to 10^0^ [21,31,33]. The samples were in triplicate, and the experiments were repeated three times. The results were the arithmetic average of triplicate experiments.

## 3. Results

### 3.1. RNase P Ribozyme Cleavage of HBV RNA Target In Vitro

Efficient ribozyme targeting demands access to interacting with its target mRNA. We used the dimethyl sulphate (DMS)-based mapping approach [23,24,37] to uncover the regions of S mRNA marked by DMS modification in cell culture, as these regions may potentially be accessible to ribozyme binding. In these experiments, human HepG2.2.15 cells harboring HBV DNA genome [21,22] were treated with DMS. Total mRNAs were collected from these cells, with the DMS-modified regions being assessed by way of primer extension assays. We selected a heavily DMS-modified position of the S mRNA as the ribozyme cleavage site.

Active and inactive M1GS ribozymes, M1-S-A and M1-S-I, were generated by joining the 3′ end of ribozymes M1 RNA and C102 RNA to a guide sequence complementary to the target S mRNA sequence, respectively. C102 RNA is an inactive ribozyme mutant originating from M1 RNA, and contains various point mutations at the P4 catalytic domain and C012 is 1 × 10^5^-fold less active at minimum compared to M1 RNA [26]. To determine the effect of an M1GS RNA that does not target S mRNA, we also included M1-P, a control ribozyme derived from M1 RNA. This ribozyme targets an mRNA essential for viral infection and replication, the mRNA encoding the protease of human cytomegalovirus (HCMV). M1-P has previously been shown to cleave the mRNA sequence coding for the protease, inhibiting the protease expression and viral infection in HCMV-infected cells [10].

In vitro, active ribozyme M1-S-A efficiently cleaved substrate s37, which included a HBV S mRNA sequence of 37 nucleotides (Table 1). Conversely, M1-S-I and M1-P hardly cleaved s37. Using kinetic analyses, we revealed the overall cleavage efficiency (i.e., (k_cat_/K_m_)^s^) of the ribozymes (Table 1). Measuring the dissociation constant (K_d_) with gel-shift assays revealed that the binding affinity of M1-S-A to substrate s37 was similar to that of M1-S-I. Thus, we used M1-S-I as the antisense effect control because this ribozyme exhibited comparable affinity to s37 as M1-S-A but could not cleave the substrate due to its mutations at the P4 active domain.

### 3.2. Ribozyme Expression in Human Cells

HepG2.2.15 cells were used as the HBV infection model system to study the effects of ribozymes on HBV gene expression and propagation in cells. These cells originated from human hepatoma HepG2 cells, containing a stable transfected full-length genome of HBV (ayw subtype). In culture, HepG2.2.15 cells produced HBV B surface (HBsAg) and e (HBeAg) antigens and engaged viral genome DNA replication [22].

**Figure 1:**
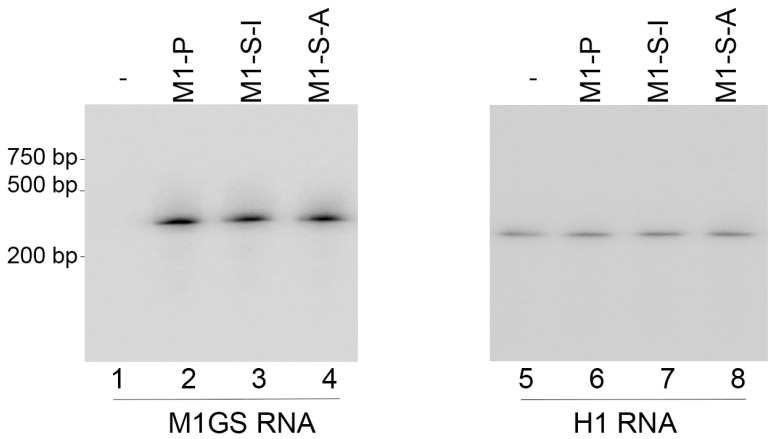
M1GS RNA expression in HepG2.2.15 cells assayed by Northern blot analyses. RNA samples (30 µg) from cells containing the empty vector pU6 (-, lanes 1 and 5), pU6-M1-P (lanes 2 and 6), pU6-M1-S-I (lanes 3 and 7), and pU6-M1-S-A (lanes 4 and 8), were separated on 2% agarose gels that contained formaldehyde, transferred to nitrocellulose membranes, and hybridized to radiolabeled probes specifically for M1 RNA (lanes 1–4) or H1 RNA (loading control) (lanes 5–8).

We cloned DNA sequences encoding ribozymes M1-S-A, M1-S-I, and M1-P into expression vector pU6, which is comprised of the small nuclear U6 RNA promoter for expressing M1GS and a green fluorescence protein (GFP) expression cassette. HepG2.2.15 cells were transfected with pU6 vector or pU6-M1-S-A, pU6-M1-S-I, and pU6-M1-P. After selection, those containing the transfected constructs were cloned. Northern blot analysis showed similar levels of ribozymes in these cells, with human H1 RNA as the loading control (Figure 1). Ribozyme expression did not exhibit substantial cytotoxicity because cells harboring the empty vector pU6 and the ribozyme-containing plasmids were indistinguishable in growth or viability for up to three months, as shown in MTT assays.

### 3.3. Ribozyme-Mediated Inhibition of HBV Gene Expression and Replication

**Figure 2:**
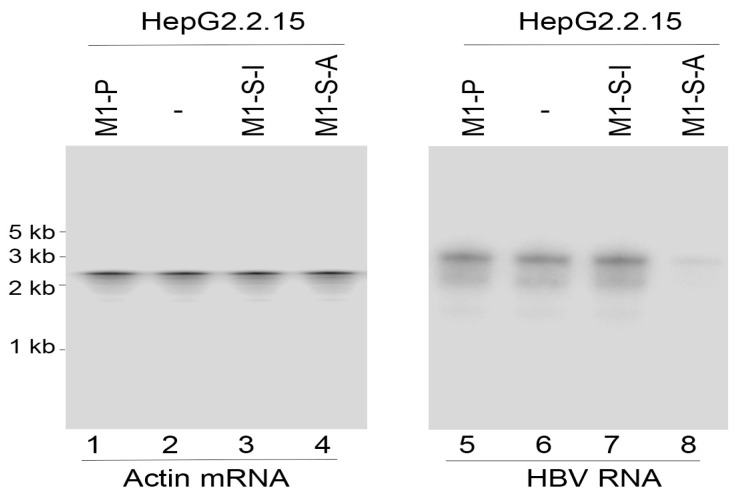
HBV transcript expression assayed by Northern blot analyses. RNA samples (25 µg) from cells containing the empty vector pU6 (-, lanes 2 and 6), pU6-M1-P (lanes 1 and 5), pU6-M1-S-I (lanes 3 and 7), and pU6-M1-S-A (lanes 4 and 8), were separated on agarose gels, transferred to nitrocellulose membranes, and hybridized to [^32^P]-radiolabeled probes specifically for the actin mRNA (loading control) (lanes 1–4) and HBV S mRNA (lanes 5–8).

**Figure 3:**
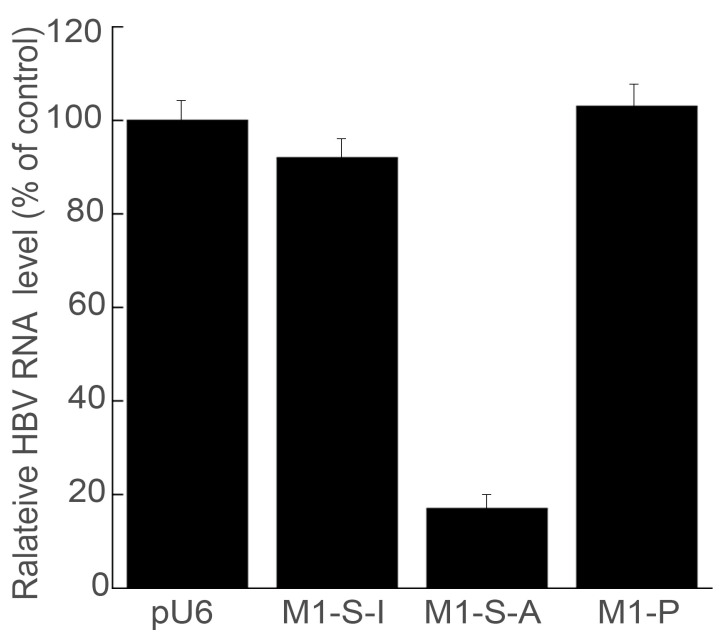
Inhibition of HBV RNA expression in HepG2.2.15 cells expressing RNase P ribozyme. RNA samples were isolated from cells and assayed for HBV RNA expression using Northern blot analysis with actin mRNA as the loading control. Experimental details are described in Materials and Methods. The values, which are the average of triplicate experiments, represent the relative percentage of the levels of HBV RNAs in cells with different ribozymes, as compared to those in cells with empty vector pU6. The error bars show the standard deviation.

We studied the HBV 3.5 kb and 2.4/2.1 kb transcript levels, which represent the pre-C mRNA/pregenomic RNA and pre-S/L mRNA, respectively [15]. We used actin mRNA as the loading control in the Northern blot experiments, since its expression is not regulated by HBV under assay conditions (Figure 2 and Figure 3) [15]. In cells expressing M1-S-A, the HBV 3.5 kb and 2.4/2.1 kb transcript levels showed ~82% reduction, compared to HepG2.2.15 cells transfected with the pU6 empty vector alone. In cells expressing M1-S-I and M1-P, however, the HBV transcript levels decreased by less than 10% (Figure 2 and Figure 3). Neither Northern blot analyses nor 5′ rapid amplification of cDNA ends (RACE) PCR assays detected any specific ribozyme-mediated cleavage products of the target RNAs. These RNA species may be rapidly degraded and thereby could not be detected since they were either lacking the 5′ capped structure or the 3′ polyA sequences.

**Figure 4:**
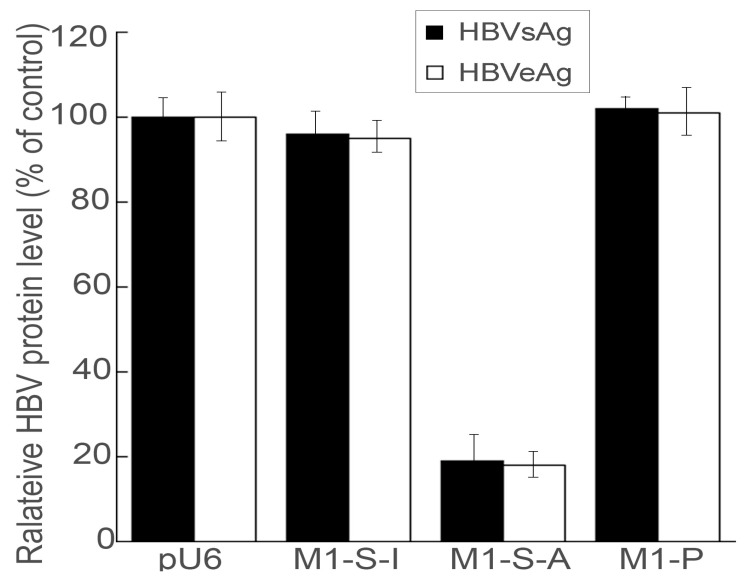
Inhibition of HBV protein expression in HepG2.2.15 cells expressing RNase P ribozyme. Media were collected from the cultures of ribozyme-expressing cells and ELISA was used to assay the HBsAg and HBeAg levels. The values, which are the average of triplicate experiments, represent the relative percentage of the levels of HBsAg and HBeAg in cells with different ribozymes, as compared to those in cells with empty vector pU6. The error bars show the standard deviation.

A decrease in HBV protein expression and production is expected due to suppression in HBV transcript levels. The levels of HBV production were assessed by studying the HBsAg and HBeAg antigen levels in cell culture supernatants [15]. The M1-S-A expressing cells exhibited a suppression of about 80% in HBsAg and HBeAg levels (Figure 4). The cells expressing M1-S-I displayed a low level of inhibition (Figure 4), likely due to an antisense effect since this ribozyme presented a comparable binding affinity to s37 as M1-S-A but could not cleave the substrate (Table 1). We observed no changes in the HBsAg and HBeAg levels in cells expressing no ribozymes or M1-P (Figure 4).

### 3.4. Ribozyme-Mediated Inhibition of HBV Genomic DNA Replication and Virus Production

**Figure 5:**
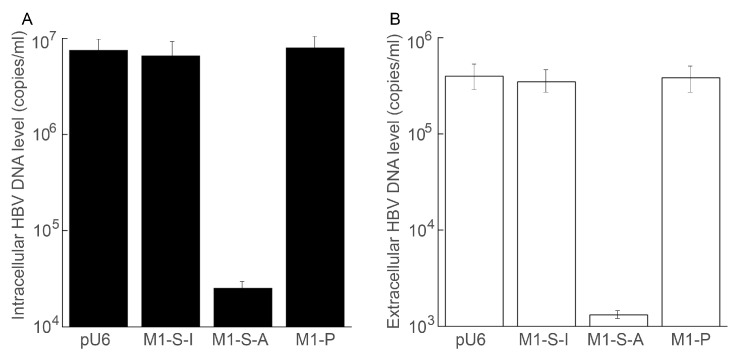
Inhibition of HBV intracellular DNA replication and virus production in HepG2.2.15 cells expressing RNase P ribozyme. HBV capsid-associated DNAs were isolated from ribozyme-expressing cells (**A**) or their culture media (**B**), and their levels were determined by qPCR. The values are the average of triplicate experiments. The error bars showed the standard deviation.

We investigated if ribozymes decreased HBV DNA replication. To achieve this, we employed quantitative PCR (qPCR) to assay intracellular capsid-associated HBV DNA levels. Cells expressing M1-S-A exhibited a decrease of 300-fold in HBV DNA levels, compared to HepG2.2.15 cells transfected with the pU6 empty vector (Figure 5A). Meanwhile, cells expressing M1-S-I or M1-CSP displayed no substantial (<10%) changes in HBV DNA levels (Figure 5A).

We also studied the effect of RNase P ribozyme targeting in regard to virus particle production. Capsid-associated viral DNA levels in culture media reflect the copy numbers of viral genomic DNA found in HBV particles released in the media. Using the qPCR approach, we assayed the levels of capsid-associated HBV DNAs in media from the cell cultures without any ribozymes or with different ribozymes. The media from cell cultures with M1-S-A exhibited a decrease of about 300-fold in HBV DNA levels, compared to that from the culture of HepG2.2.15 cells with the pU6 empty vector (Figure 5B). The cell culture media with M1-S-I or M1-P, however, displayed no substantial (<10%) changes in HBV DNA levels (Figure 5B). These results indicate a suppression of both HBV genome DNA replication and particle production in M1-S-A expressing cells.

## 4. Discussion

The RNase P ribozyme technology is a promising strategy for gene targeting because it employs M1GS RNA to cleave a target RNA [3,38]. Prior to this work, there were no reports of RNase P-based ribozyme inhibiting HBV gene expression and production in human cells. Here, we showed that an engineered RNase P ribozyme, M1-S-A, efficiently cleaved the S mRNA sequence in vitro. HepG2.2.15 cells with M1-S-A exhibited a substantial suppression in the levels of HBV RNAs, proteins, genomic DNA, and virus production, compared to HepG2.2.15 cells that were transfected with the pU6 empty vector and did not express any RNase P ribozymes. Cells with M1-S-I and M1-P, however, displayed no substantial changes in those same parameters. M1-P targeted the mRNA encoding the HCMV protease and did not cleave the S mRNA sequence in vitro. M1-S-I exhibited no catalytic activity due to P4 domain mutations but displayed comparable affinity to the S mRNA sequence in vitro as M1-S-A. Therefore, these observations indicated that the anti-HBV effect in cells expressing M1-S-A is due to the catalytic slicing of the S mRNA by this ribozyme and is not due to the antisense effect of the guide sequence or other nonspecific effects from the ribozymes.

Multiple lines of evidence presented here imply M1GS RNAs to be active and suppress HBV replication in cultured cells. First, the presence and expression of M1GS sequences did not substantially alter the capability and growth of HepG2.2.15 cells for up to three months. Second, the ribozymes appeared to be active in cleaving the S mRNA in cultured cells. Decreases in both HBV gene expression and viral DNA replication were observed in cultured cells that were transfected with pU6-M1-S-A but not control constructs pU6, pU6-M1-S-I, or pU6-M1-P. Third, the magnitude of decrease in the HBV transcript levels in cultured cells correlated well with that in the HBsAg/HBeAg and HBV DNA levels (Figure 2, Figure 3, Figure 4 and Figure 5). Hence, the M1-S-A-mediated antiviral effect (e.g., inhibition of viral protein expression and genomic DNA synthesis) appears to originate from the suppression of HBV transcript expression due to the ribozyme-mediated slicing of the S RNA.

In our previous studies, customized designed external guide sequences (EGSs) were constructed and introduced into mouse cells and human HepG2.2.15 cells to induce endogenous mouse and human RNase P, respectively, to cleave HBV RNA sequences and inhibit HBV gene expression and replication [36,39]. In the current study, we used a different approach by constructing M1GS ribozymes in which M1 RNA (the catalytic RNA subunit of RNase P in *E. coli*) is linked to a guide sequence targeting a different region of the S mRNA sequence. M1GS ribozymes were introduced as exogenous gene-targeting agents to degrade the target HBV RNAs. Because of this, the M1GS ribozyme can be used to target both cytoplasmic and nuclear mRNAs while the EGS-mediated gene targeting action to downregulate gene expression is believed to work in the nuclei where human RNase P is primarily localized [38]. Moreover, the M1GS ribozyme is different from human RNase P, which contains at least 10 different protein subunits and a RNA subunit (H1 RNA), which is hardly catalytically active in the absence of the protein subunits [3,40].

As an anti-HBV therapeutic, the M1GS ribozyme approach may have several potential limitations and issues that should be addressed. First, M1GS RNA needs to be delivered to specific types of tissues and cells such as the liver and hepatocytes. New gene-transfer methods, including liposome-based or viral vector-based agents, are in development and have shown great promise for the efficient delivery of exogenous therapeutic gene-expression cassettes to different tissues and cells in vivo. These reagents can be used to deliver the expression cassettes of M1GS RNAs into the liver and hepatocytes.

Second, RNase P ribozymes with better targeting activities should be developed. In vitro selection procedures have been documented to be capable of generating highly active and functional RNA molecules, including ribozymes, with enhanced activity [41,42,43]. We have used these procedures to produce RNase P ribozyme variants more capable of cleaving an mRNA compared to the ribozyme derived from the wildtype M1 RNA sequence [44]. When expressed in cultured cells, these ribozyme variants led to more inhibition of the herpes simplex virus (HSV) and human cytomegalovirus (HCMV) gene expression and better reduction in HCMV infection and replication compared to the ribozyme derived from the wild-type M1 RNA sequence [25,45]. Further studies of engineered M1GS ribozymes and their delivery with newly constructed delivery vectors should help develop these ribozymes for anti-HBV applications.

Another potential limitation is the potential off-target effects of the M1GS ribozyme. Little is currently known about M1GS sequence specificity as a gene targeting agent. Additional studies are needed to investigate the sequence specificity and potential cytotoxicity of M1GS ribozymes in a variety of human cells and tissues as well as in animal models. These studies will reveal the safety profiles of RNase P ribozyme for its use in potential clinical applications. As observed in previous studies using mouse and human RNase P-associated EGSs, effective inhibition of HBV gene expression and replication was found in M1GS ribozyme-expressing cells in the current study. Our study here raises exciting prospects for generating M1GS ribozymes for anti-HBV therapy. 

There are currently several compounds that have been approved by the FDA for treatment of HBV infection and diseases [13,15]. These compounds are effective in controlling viral infection but could not completely eliminate HBV chronic infection. One major limitation of these currently FDA-approved treatments available against HBV infection is the inability of these compounds to remove the viral covalently closed circular DNA (cccDNA) essential for HBV chronic infection and persistence [46]. RNase P ribozymes also have a similar limitation because they only slice HBV RNAs and may not target viral cccDNA. It will be worth studying whether there is a beneficial or synergistic anti-HBV effect, when RNase P ribozymes are combined with anti-HBV cccDNA approaches or drugs [47]. These investigations will help in establishing more effective treatments for HBV infection and its associated diseases.

## Figures and Tables

**Table 1:** RNase P ribozyme cleavage of substrate s37 (overall cleavage rates ((k_cat_/K_m_)^s^) and binding dissociation constants (K_d_)). Experimental details are described in Material and Methods.

Enzyme	(k_cat_/K_m_)^s^ (µM^−1^·min^−1^)	K_d_ (nM)
M1-S-A	0.35 ± 0.09	0.40 ± 0.07
M1-S-I	<5 × 10^−5^	0.38 ± 0.07
M1-P	<5 × 10^−5^	ND ^1^

^1^ The means from three independent experiments are shown. “ND” not determined.

## Data Availability

Not applicable.
